# Identification and validation of a novel ubiquitination-related gene *UBE2T* in Ewing’s sarcoma

**DOI:** 10.3389/fonc.2023.1000949

**Published:** 2023-02-16

**Authors:** Guoxin Qu, Yuanchun Xu, Ye Qu, Jinchao Qiu, Guosheng Chen, Nannan Zhao, Jin Deng

**Affiliations:** ^1^ Department of Orthopaedics, The First Affiliated Hospital of Hainan Medical University, Hainan Medical University, Haikou, China; ^2^ Department of Emergency, The Affiliated Hospital of Guizhou Medical University, Guizhou Medical University, Guiyang, China; ^3^ Department of General Surgery, State Key Laboratory of Trauma, Burns and Combined Injury, Research Institute of Surgery, Daping Hospital, Army Medical University, Chongqing, China; ^4^ Department of Trauma Surgery, The Second Affiliated Hospital of Hainan Medical University, Hainan Medical University, Haikou, China; ^5^ Department of Ophthalmology, The First Affiliated Hospital of Hainan Medical University, Hainan Medical University, Haikou, China

**Keywords:** *UBE2T*, ubiquitination, Ewing’s sarcoma, prognosis, diagnosis, biomarker

## Abstract

**Background:**

Ewing’s sarcoma (ES) is one of the most prevalent malignant bone tumors worldwide. However, the molecular mechanisms of the genes and signaling pathways of ES are still not well sufficiently comprehended. To identify candidate genes involved in the development and progression of ES, the study screened for key genes and biological pathways related to ES using bioinformatics methods.

**Methods:**

The GSE45544 and GSE17618 microarray datasets were downloaded from the Gene Expression Omnibus (GEO) database. Differentially expressed genes (DEGs) were identified, and functional enrichment analysis was performed. A protein–protein interaction (PPI) network was built, and key module analysis was performed using STRING and Cytoscape. A core-gene was gained and was validated by the validation dataset GSE67886 and immunohistochemistry (IHC). The diagnostic value and prognosis evaluation of ES were executed using, respectively, the ROC approach and Cox Regression.

**Results:**

A total of 187 DEGs, consisting of 56 downregulated genes and 131 upregulated genes, were identified by comparing the tumor samples to normal samples. The enriched functions and pathways of the DEGs, including cell division, mitotic nuclear division, cell proliferation, cell cycle, oocyte meiosis, and progesterone-mediated oocyte maturation, were analyzed. There were 149 nodes and 1246 edges in the PPI network, and 15 hub genes were identified according to the degree levels. The core gene (*UBE2T*) showed high expression in ES, validated by using GSE67886 and IHC. The ROC analysis revealed *UBE2T* had outstanding diagnostic value in ES (AUC = 0.75 in the training set, AUC = 0.90 in the validation set). Kaplan-Meier (analysis of survival rate) and Cox Regression analyses indicated that UBE2T was a sign of adverse results for sufferers with ES.

**Conlusion:**

*UBE2T* was a significant value biomarker for diagnosis and treatment of ES, thereby presenting a novel potential therapeutic target for ES as well as a new perspective for assessing the effect of treatment and prognostic prediction.

## Highlights

1. Ewing’s sarcoma (ES)-related DGEs were verified ground on the GEO database and TCGA database.
2. In all 187 DEGs and 15 hub genes were closely associated with the progression of ES.
3. One key alteration gene (*UBE2T*) was differently expressed between tumor specimens and normal specimens, suggesting that this gene may be a latent prognosis predictor for ES stufferers.
4. Validation set and IHC confirmed that the UBE2T was overexpressed in ES but not in normal tissues.
5. In patients with ES, *UBE2T* can be used as a biomarker with important diagnostic value as well as an independent prognosis. The discovery of *UBE2T* will provide a new perspective for ES research.

## 1 Introduction

Ewing sarcoma (ES), an invasive ossature and soft-tissue cancer, is a frequent malignant bone tumor, ranking second among the pediatric population, and it also affects adolescents (1, 2). Presently, the standard of treatment for ES involves multimodal therapy, including surgical resection, local radiation therapy, and intensive multiagent chemotherapy (3). Despite tremendous advances in diagnosis, treatment, and prognosis of this illness with the advancement of medicine, nonspecific clinical features of ES give rise to symptoms that are unremarkable in the early stages, and high metastasis and recurrence rates have become the main poor outcomes of treatment(2, 4, 5). Furthermore, as the complete mechanisms of the molecular pathology for ES tumorigenesis and progression are unknown, there are few efficacious ways available to early diagnose the disease, resulting in a high mortality rate and death rate. As a result, successfully implementing diagnosis and treatment approaches requires a thorough insight into the mechanisms of the molecular biology underlying tumorigenesis, multiplication, and recurrence of ES.

**Figure 1:**
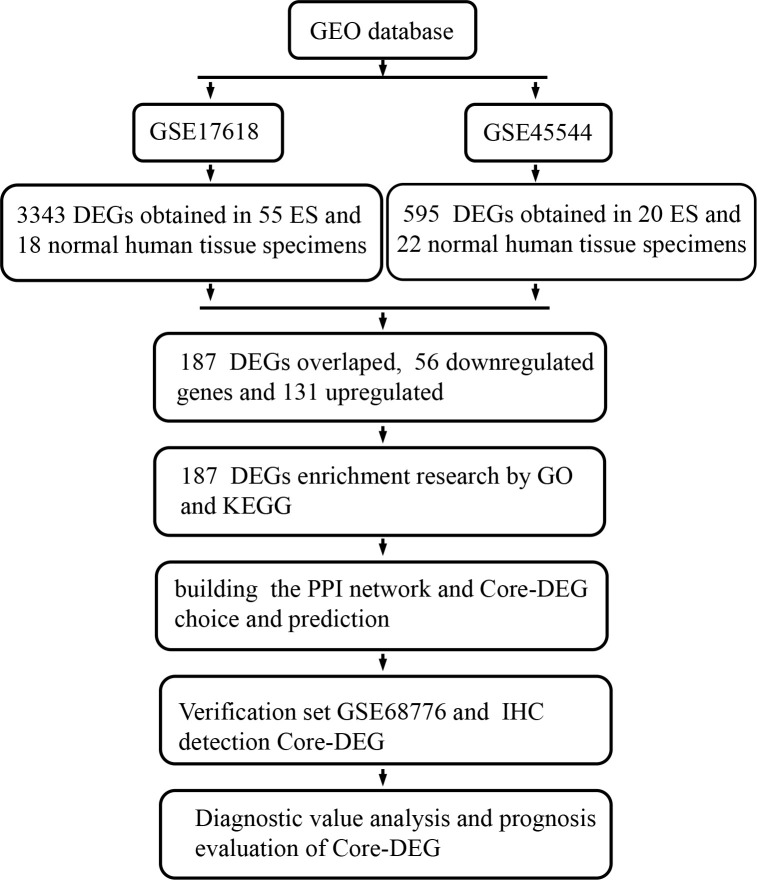
This study’s flow diagram. GEO, Gene Expression Omnibus database; ES, Ewing’s sarcoma; DEGs, Differentially expressed genes; GO, Gene Ontology; KEGG, Kyoto Encyclopedia of Genes and Genomes; PPI, protein-protein interaction; IHC, Immunohistochemical.

Affymetrix techniques and bioinformatics research have been increasingly employed to monitor gene expression levels in recent decades, allowing for the efficiently identification of DEGs and functional pathways related to the tumorigenesis and development of ES. There are many microarray data sets shared and kept in accessible web databases. In order to the screening of additional molecular markers, many microarray data information for identifying ES genes can be available from the database. To evaluate DEGs between tumor specimens and nontumor specimens, two microarray datasets collected from the GEO (Gene Expression Omnibus) (6) data bank were obtained and processed in this study. And to research the latent functions of these DEGs, we applied GO (Gene Ontology) (7, 8), KEGG (Kyoto Encyclopedia of Genes and Genomes) (9) pathway enrichment study, and PPI (protein–protein interaction) network research. Finally, the current investigation discovered a total of 187 DEGs, 15 hub genes and 1core DEGs, and further validation experiments, diagnostic value and prognosis analysis were carried out on core-DEGs, which discovered a valuable latent biomarker for the diagnosis, remedy, and prognosis evaluation of ES (**Figure 1**).

## 2 Materials and methods

### 2.1 Microarray data

This currently study obtained two training datasets, the GSE17618 (10) and GSE45544 (11) from the GEO data bank (http://www.ncbi.nlm.nih.gov/geo) (6), which is a publicly accessible functional genetic and genomic data repository for high-throughput gene expression information, chips, and microarrays. The GSE45544 dataset (including 20 ES and 22 noncancerous tissue specimens) is dependent on Affymetrix GPL6244 platform data (Affymetrix Human Gene 1.0 ST Array), whereas the GSE17618 dataset (73 specimens, ES n = 55 and normal n = 18) is built on Affymetrix GPL570 platform data (Affymetrix Human Genome U133 Plus 2.0 Array). Furthermore, the GSE68776 (12) from the GPL570 platform (Affymetrix Human Exon 1.0 ST Array) was extracted as a validation dataset (ES specimens n = 32; normal n = 33) to be used later.

### 2.2 Identification of DEGs

The GEO2R (http://www.ncbi.nlm.nih.gov/geo/geo2r) was executed to pick out the DEGs between ES and noncancerous specimens. GEO2R is a web-based interactive tool that allows clients to gain data by comparing two or more GEO series datasets to discover DEGs from experimental results, to analyze DEGs, and to determine highly expressed and negatively regulated DEGs between ES samples and normal specimens. And, the adjusted P values (adj. P) and Benjamini and Hochberg false discovering rates were employed to provide a balance between the excavation of statistically meaningful genes and the restrictions of false-positives. Probe sets with no associated gene symbols were eliminated, as were genes with multiple probe sets. |logFC (fold change)| ≥ 2 and adj. P values < 0.01 were deemed statistically meaningful.

### 2.3 DEG enrichment research using GO and KEGG

The Databank for Annotation, Visualization, and Integrated Discovery (DAVID; http://david.ncifcrf.gov) (6.8 version)(13) is a publicly viewable laboratory biological information data bank that incorporates analytical and statistical tools based on biological analysis and offers a wide range with a suite of integrated functional annotation data of proteins and genes to continue investigating biological data information. GO is a computer-based bioinformatics software that is mostly used to annotate genes and research their biological processes (7). KEGG is a computer statistical resource database that evaluates high-standard biological processes and function systems from a wide range of molecular datasets and discovers pathways in which DEGs may play a major role (14). The DAVID online information system was implemented for the functional study of DEG biology. P < 0.05 was accepted as statistical significance.

### 2.4 Building and analyzing of the PPI network and module

The PPI network was built utilizing the STRING (Search Tool for the Retrieval of Interacting Genes, http://string-db.org) (11.0 version) (15) online database. Assessing and analyzing protein-protein interactions may critically reveal the mechanisms of the generation or progression of illnesses. An interaction with a combined score > 0.4 was considered statistically significant. Cytoscape (3.8.2 version) is an available open-source bioinformatics software tool utilized to visualize network systems of molecular interaction (16). And, the MCODE (Molecular Complex Detection) (2.0 version) plugin of Cytoscape is an application (APP) software used to search densely connected regions in large PPI networks (17) and to verify the most major module section (MCODE-DEGs). the following conditions for filtering were used: MCODE scores are greater than 5, the degree cutoff is 2, the node score cutoff is 0.2, the maximum depth is 100, and the k-score is 2,. and the biological process investigation was carried out with Cytoscape ClueGO (18) (version 2.5.8). Next, a hierarchical clustering (using R the pheatmap package) of MCODE-DEGs was implemented based on the expression profiling of training datasets.

### 2.5 Core-DEG choice and verification set detection

The degree levels in the cytoHubba (19) Cytoscape plugin were implemented to define the hub genes. Cytoscape ClueGO (18) (version 2.5.8) was used to depict the biological process investigation of core genes. Furthermore, mutant survival, including overall survival and illness-free survival, was assessed to further screen the hub gene core-DEG employing Kaplan-Meier methods in the cBioPortal web tool (http://www.cbioportal.Org) (20). Then, GSE68776 (ES n = 32, control n = 33) was used to validate the expression of the core-DEG, which was depicted in the volcano plot by the “ggplot2” software.

### 2.6 Immunohistochemistry experiment

A total of 11 paraffin-embedded Ewing’s sarcoma tissues (8 males (72.73%) and 3 females (27.27%)) were provided by Daping Hospital (Chongqing, China). All patients signed a written informed consent form. 3mm tumor paraffin sections were blocked for 1 hour at room temperature with sheep serum blocking solution (Zhongshan Jinqiao, China), then diluted 1/100 with anti-UBE2T antibody and anti-CD99 antibody(Cohesion Biosciences, UK) at 4 °C overnight. Then, for 2 hours at room temperature, goat anti rabbit secondary antibody (1:200 dilution; Biyuntian, China) was administered for color development (Zhongshan Jinqiao, China), and the nucleus was stained with hematoxylin. The results were then examined under an optical microscope (Ningbo Konfoong, China). Besides, to assess the area and density of stained regions, as well as the internal grating optical density (IOD) values of IHC sections, Image Pro Plus version 6.0 software (Media Cybernetics, Rockville, MD, USA) was employed. The signal density of a tissue region chosen at random from five locations was counted and statistically assessed using a blind approach.

### 2.7 Diagnostic value analysis of *UBE2T* in the ES

The receiver operating characteristic curve (ROC) technique in the Python package was executed to analyze core gene diagnostic effectiveness according to the training set and validation set.

### 2.8 Identification of DEGs subgroups in ES

To better understand the biological phenotype of MCODE-DEGs regulation in the tissue of ES patients, the MCODE-DEGs based on gene expression profiles in the training dataset were grouped using Consensus Cluster Plus (21). UMAP (version 0.2.7.0; a R software tool) was used to do dimension reduction analysis. Following that, the Python R package was used to do a visual analysis of the heat map and boxplot of the differential expression of MCODE module genes. Finally, Kaplan Meier method was used for survival analysis to obtain the most significantly different subgroups.

### 2.9 The Cox regression analyses of core-DEG

Based on expression profiles, using the R language Python module, a raincloud diagram (22) is utilized to graphically assess core-DEG expression differences in C3 and C4 subgroups. Then, the Cox regression analysis was used to further evaluate the relationship between the core-DEG expression and prognosis using the R software package survival and Maxstatat, and the best cutoff risk score was calculated. In addition, the Python package was used to investigate the association between various risk scores, patients’ survival time, status, and gene expression changes.

### 2.10 Statistical analysis

For statistical analysis, R package (version 4.0.2), IBM SPSS 26.0 software and graphpad prism 8 (graphpad Software Inc, CA, USA) were utilized. All data is provided as the means ± standard deviation(SD). The Student’s t test and Wilcoxon rank sum test were conducted to see if there were any differences between the sample groups. For survival analysis, the Kaplan-Meier technique was applied. Furthermore, ROC technology was adopted to assess the diagnostic effectiveness of core gene, which was represented by the Area Under Curve (AUC). The sensitivity and specificity of the gene were calculated. When the Youden’s index was adjusted to its maximum value, the optimum gene cut-off value was attained. Later, the prognosis analysis was examined using Cox regression. *P*<0.05 was considered statistically significant.

## 3 Results

### 3.1 Identification of DEGs in ES

**Figure 2:**
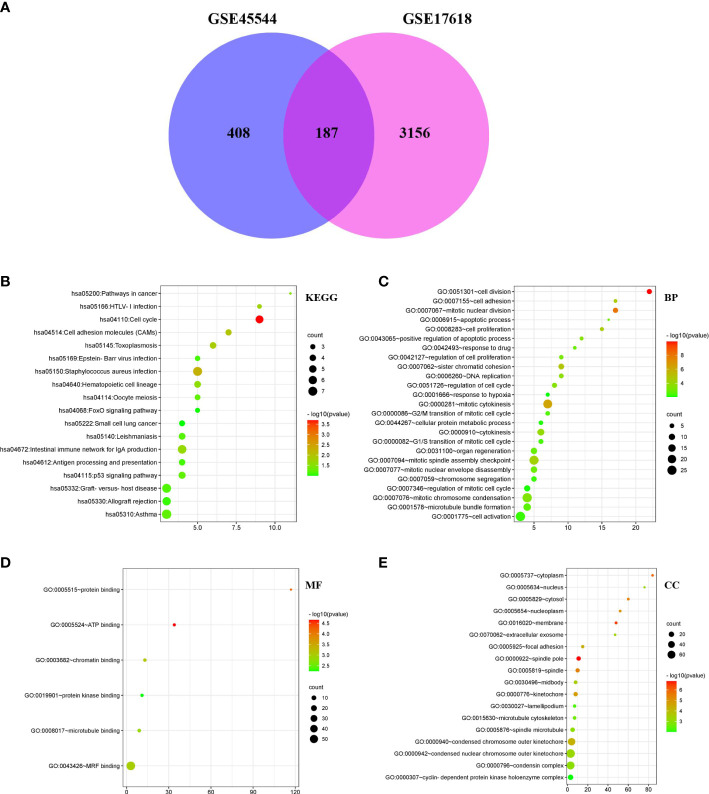
DEG Venn diagram; Bubble Plot of the GO and KEGG enrichment study for DEGs. **(A)** DEGs in the GSE17618 and GSE45544 mRNA expression profiling datasets were filtered with a fold change > 2 and a *P* value < 0.01. 187 genes overlapped between the two datasets. **(B–E)** The P value is shown by the progressively shifting hue, and the quantity of genes is denoted by the size of the black dots.

After standardizing the microarray findings, DEGs (595 in GSE45544 and 3343 in GSE17618) were discovered. According to the Venn diagram, the overlapping section of the two datasets included 187 genes (**Figure 2A**). There were 56 downregulated genes and 131 upregulated genes in the comparison of Ewing sarcoma tissues and noncancerous tissues (**Table 1**).

**Table 1:** Analyzation of the datasets identified 187 DEGs, including 131 upregulated and 56 downregulated genes, in tumor samples.

Status	DEGs
Upregulated	*CD44 TPX2 CCNB1 TTC37 FRY GINS1 A2M SLCO2B1 ANLN LYZ BCLAF1 FOXM1 CHST15 PLIN2 CDC6 CST3 HOXD13 IFI16 ZNF146 RDX BDP1 SPDL1 CDK4 TYMS MELK ECT2 SNCA CDH11 NUF2 PDK4 STMN1 UBE2T CKS2 FAM84B KIF23 C1S PDLIM1 DKK3 ANKH NDRG1 MYLK CCNB2 MCM7 PRC1 CENPI JPH1 EPAS1 PMP22 KIAA0101 HLA-DPB1 BHLHE40 CELF2 NUP107 DLGAP5 MKI67 TM4SF1 PLPP3 PTPRM TOP2 EXO1 PDGFRA BHLHE41 IGK///IGKC SAT1 YPEL2 HSPA1B///HSPA1A MEF2C OAT CHPT1 VAMP8 FGL2 SQRDL ZNF704 CCT2 PAPPA SMC4 GUCY1B3 CKS1B TEAD2 GSN RHOB HLA-DPA1 EBF3 FBXO5 ZNF644 TICRR PBK PRR11 TXNIP HEATR1 ITGB3BP PRPF40A SKA3 DPT TYROBP SMC2 ASPM ATAD2 WDHD1 METTL7A BUB1B DTL TGFBR2 JAK1 LAPTM5 FAM114A1 KIF20A KCTD12 CDC5L NCAPG PLK1 RFTN1 ATP1B1 TNFSF10 CHEK1 CRYAB KIF11 SLC40A1 CD9 RFC4 TPR BUB1 BRIP1 CAD CKAP2 CXCL12 AMICAL2 FANCI CENPF NUSAP1 IGF2BP1*
Downregulated	*ABHD2 CD53 SORBS2 ALDH6A1 SUSD6 ITGA6 TPPP3 S100A16 SLC22A3 ADGRG1 MAN1A1 CECR1 RHOU SELPLG MGST2 TNFRSF21 SRPX IL10RA ENTPD1 PRELP SATB1 SYNPO2 DNAJA4 TSC22D3 RCAN2 NUPR1 TAPBP PLXNC1 TOB1 MYH11 C10orf10 LRP10 DOCK9 TLE1 MGLL CD59 PTGDS PXDC1 PEA15 SERPINB1 SERPINB6 WIPI1 RNF144B ENDOD1 ATP8A1 CA2 PPFIBP2 HLA-DMA GAS7 NEDD9 ITGA9 CTSZ CSF1R APBB1IP FRMD4B PIK3IP1*

### 3.2 DEG enrichment analysis utilising GO and KEGG

DAVID was carried out to accomplish function and passage enrichment research to ascertain the biology classification of DEGs. The results were visualized using the R language pack 4.1.3 version. The DEGs were considerably enriched in the cell cycle and Staphylococcus aureus infection, according to analyzation of the KEGG pathway (**Figure 2B**). According to GO analysis, alterations to BPs (biological processes) in DEGs were primarily enriched in cell division, cell proliferation, cell adhesion, mitotic nuclear division, positive regulatory process of apoptosis, and drug response (**Figure 2C**). ATP bound, protein bound, chromatin bound, and protein kinase bound were considerably enriched in the DEGs’ molecular functions (MFs) (**Figure 2D**). DEGs’ CC (cell component) alterations were primarily enriched in the spindle pole, membrane, cytoplasm, focal adhesion, cytosol, nucleoplasm, extracellular exosome, and nucleus (**Figure 2E**).

### 3.3 Building and analyzing of the PPI network and module

**Figure 3:**
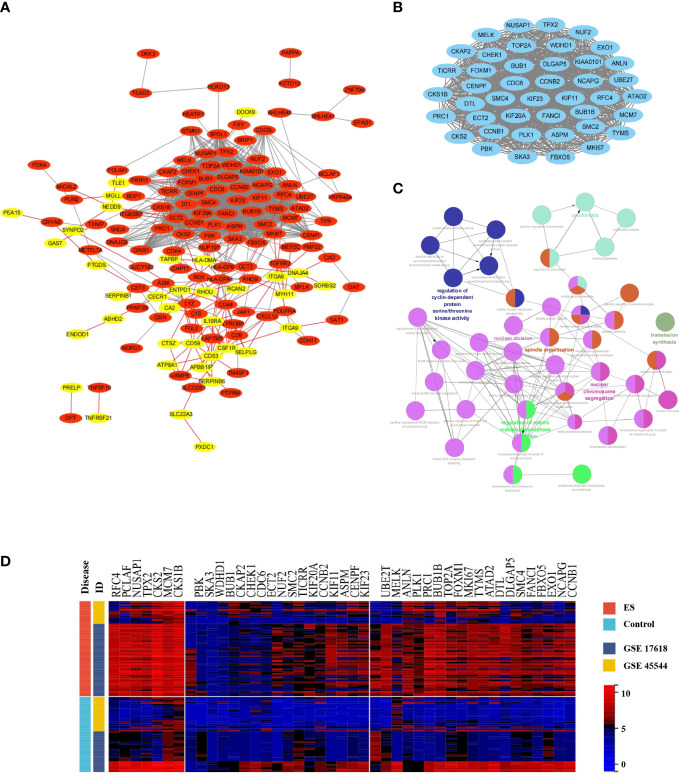
Construction of the PPI network, and MCODE module; Biology process analyzation and hierarchical clustering of MCODE-DEGs. **(A)** The Cytoscape program was built to obtain the DEGs PPI network. Genes upregulated were highlighted in light red, whereas genes downregulated were highlighted in light yellow. **(B)** The PPI network yielded the most significant module (MCODE-DEGs), having 43 nodes and 857 edges. **(C)** ClueGO was employed to evaluate the biological processes of the MCODE-DEGs *P* < 0.01 was judged statistically meaningful. The node’s dark hue represented the rectified P value of ontologies. The quantity of genes participating in ontologies was represented by node’s size. **(D)** The heat map demonstrated significantly different in expression levels of MCODE-DEGs between the ES and control group. Red represented upregulation of genes; Blue represented downregulation.

Then, MCODE, a Cytoscape plugin tool, was executed to establish the most meaningful module of the DEG PPI network. The PPI network (**Figure 3A**) included 149 nodes and 1246 edges, with 36 genes down-regulated and 113 genes up-regulated, whereas the MCODE network (**Figure 3B**) was composed of 43 nodes and 857 edges. Furthermore, the biological process analysis of MCODE-DEGs was visualized by Cytoscape ClueGO (**Figure 3C**), which was concentrated on regulation of cyclin-dependent proteins, serine/threonine kinase activity, cytokinesis, nuclear chromosome segregation, regulation of mitotic metaphase/anaphase transition, and spindle organization. Besides, Hierarchical clustering discovered that the genes expression level of the most important module significantly distinguished the ES samples from the nontumorous samples according to the expression profiles of training sets (**Figure 3D**).

### 3.4 Core-DEG choice and evaluation

**Figure 4:**
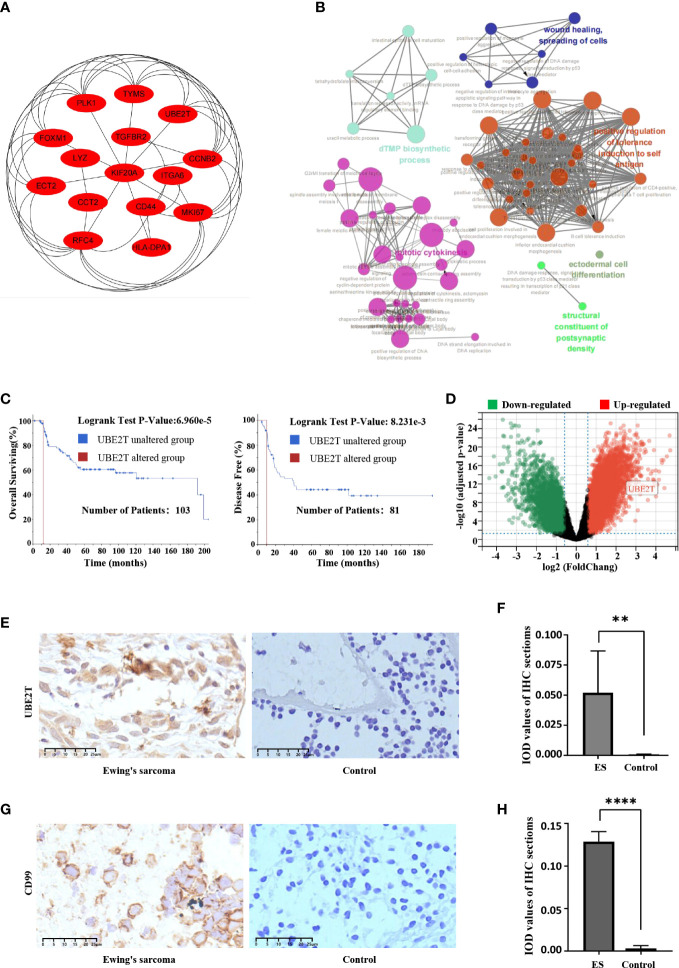
The hub genes’ connection network and biological process research; Core-DEG obtained by the cBioPortal web and verified by the GSE68776 and IHC. **(A)** The hub genes were obtained by CytoHubba with 15 nodes and 43 edges. **(B)** ClueGO was utilized to examine the biological processes of hub genes. The node’s dark hue represents the rectified *P* value of ontologies. The quantity of genes participating in ontologies is represented by node’s size. *P* < 0.01 was judged statistically meaningful. **(C)** The cBioPortal official website was employed to complete overall surviving and illness-free surviving studies of core-DEG, *P* < 0.05. **(D)** The volcanic plot showed expression of *UBE2T* in validation set GSE68776, with |log2FC|≥1.5, adjusted *P*<0.05. **(E–H)** The IHC findings revealed that the *UBE2T* protein **(E, F)** and CD99 **(G, H)** were overexpressed in ES but not in normal tissues, and the Student’s t test showed significant differences. (**P<0.01, ****P<0.0001).

The first fifteen hub genes, which included *CCNB2*, *CCT2*, *CD44*, *ECT2*, *FOXM1*, *HLA-DPA1*, *ITGA6*, *KIF20A*, *LYZ*, *MKI67*, *PLK1*, *RFC4*, *TGFBR2*, *TYMS*, and *UBE2T*, were defined with the degree levels in the cytoHubba Cytoscape plugin, and an interaction network of the hub genes was constructed, resulting in 15 nodes and 43 edges (**Figure 4A**). Meanwhile, **Table 2** lists the names, descriptions, and roles of these hub genes. Then, the Cytoscape ClueGO software was employed to investigate the biological processes of hub genes, which were primarily concentrated on Mitosis cytokinesis, the dTMP biological process, and the positive regulation of self-antigen tolerance induction; these data imply that hub genes have a significant function in regulating the cell cycle and homeostasis in the internal environment (**Figure 4B**). Furthermore, the mutated survival analyses of the hub genes was accomplished in cBioPortal online using Kaplan-Meier method. Among the 15 hub genes, only the survival analysis of *UBE2T* with and without alteration by the log-rank test demonstrated a statistically meaningful (P < 0.05) *UBE2T* alteration showed a significant lower overall and illness-free survival (**Figure 4C**), and had a poorer outcome. These data suggest that *UBE2T* may be an important biomarker in the progression of ES. As a result, *UBE2T* was defined as the “core-DEG,” which will be studied later.

**Table 2:** Functional annotation of 15 hub genes selected by cytoHubba.

No.	Gene name	Whole name	Function
1	*CCNB2*	G2/mitotic-specific cyclin-B2	Member of the cell cycle family and is needed for cyclin regulation during the G2/M (mitosis) transition. Sub-family of cell cycle AB
2	*CCT2*	T-complex protein 1 sub-unit beta	Molecularly chaperone; aids in protein folding after ATP hydrolysis. As a component of the BBS/CCT complex, it may have a function in the formation of BBSome, a compound related to ciliogenesis that regulates vesicle transport to the cilia.
3	*CD44*	CD44 antigen	hyaluronic acid receptor (HA). Its affinity for HA, as well as its affinity for other ligands including osteopontin, collagens, and matrix metalloproteases, mediates cell-cell and cell-matrix interactions (MMPs).
4	*ECT2*	Protein ECT2	guanine nucleotide exchange factor (GEF) that catalyzes the conversion of GDP to GTP. boosts guanine nucleotide swap on Rho family small GTPase members such as RHOA, RHOC, RAC1, and CDC42.
5	*FOXM1*	Forkhead box protein M1	Transcriptional factor that regulates the expression of cyclin genes that are needed for DNA replication and mitosis.
6	*HLA-DPA1*	HLA class II histocompatibility antigen, DP alpha 1 chain	Bounds peptides produced from antigens and displays them on the cell face for identification by CD4 T-cells *via* the endocytic pathway of antigen presentation cells (APC).
7	*ITGA6*	Integrin alpha-6	Platelets have an alpha-6/beta-1 integrin receptor for laminin. Integrin alpha-6/beta-4 is a laminin receptor in epithelium cells and performs an important structural function in the hemidesmosome (By similarity).
8	*KIF20A*	Kinesin-like protein KIF20A	Mitosis kinesin is needed for cytokinesis regulated by the chromosomal passenger complex (CPC). Following PLK1 phosphorylation, implicated in PLK1 recruitment to the central spindle.
9	*LYZ*	Lysozyme C	Lysozymes are principally bacteriolysis enzymes.
10	*MKI67*	Proliferation marker protein Ki-67	After nucleal envelope destruction, this protein is needed to maintain individual mitosis chromosomes disseminated in the cytoplasm.
11	*PLK1*	Serine/threonine-protein kinase PLK1;	Serine/threonine protein kinase that regulates spindle assembly and centrosome maturity, the remove of cohesins from chromosomal arms, the deactivation of anaphase-promoting complex/cyclosome (APC/C) regulators, and the control of mitosis and cytokinesis.
12	*RFC4*	Replication Factor C subunit 4	The auxiliary proteins proliferation cell nucleal antigen (PCNA) and activator 1 are needed for the elongation of primed DNA examples by DNA polymerase delta and epsilon.
13	*TGFBR2*	TGF-beta receptor type-2	a transmembrane serine/threonine kinase that interacts with TGFBR1, the nonpromiscuous receptor for the TGF-beta cytokines TGFB1, TGFB2, and TGFB3.
14	*TYMS*	Thymidylate synthase	Adds to the route of *de novo* mitochondrion thymidylate biosynthesizing
15	*UBE2T*	Ubiquitin-conjugating enzyme E2T	It receives E1 compound ubiquitin and catalyzes its covalently binding with other proteins. Monoubiquitination is catalyzed. Mitomycin-C (MMC)-induced DNA restore. Through interaction with the E3 ubiquitin-ligase FANCL and catalytic mono-ubiquitination of FANCD2, it acts as a particular E2 ubiquitin-ligase for the Fanconi anemia complex.

### 3.5 Expression change of UBE2T and CD99 in the validation data set and IHC of ES samples

The expression of core-DEG was validated using GSE68776. The volcanic plot displayed that 15,440 DEGs were found (up = 939, down = 6,435; |log2FC|≥1.5; adjusted *P*<0.05). *UBE2T* was significantly upregulated in validation data sets (**Figure 4D**). Besides, IHC was used to identify the expression of UBE2T protein in Ewing’s sarcoma and normal tissues. The findings revealed that the UBE2T protein was overexpressed in ES but not in normal tissues (**Figure 4E**). There was a statistically significant difference between the groups (*P*<0.01) (**Figure 4F**). Obviously, the research data supported our prediction. Furthermore, CD99 has a high specific diagnostic value in IHC of ES tissue, so it is necessary to observe the difference between the ES sample and the control group. IHC results showed that CD99 was diffusely positive on the cell membrane of ES tissue (**Figure 4G, H**).

### 3.6 Diagnostic performance of *UBE2T* in the ES training set and verification set

**Figure 5:**
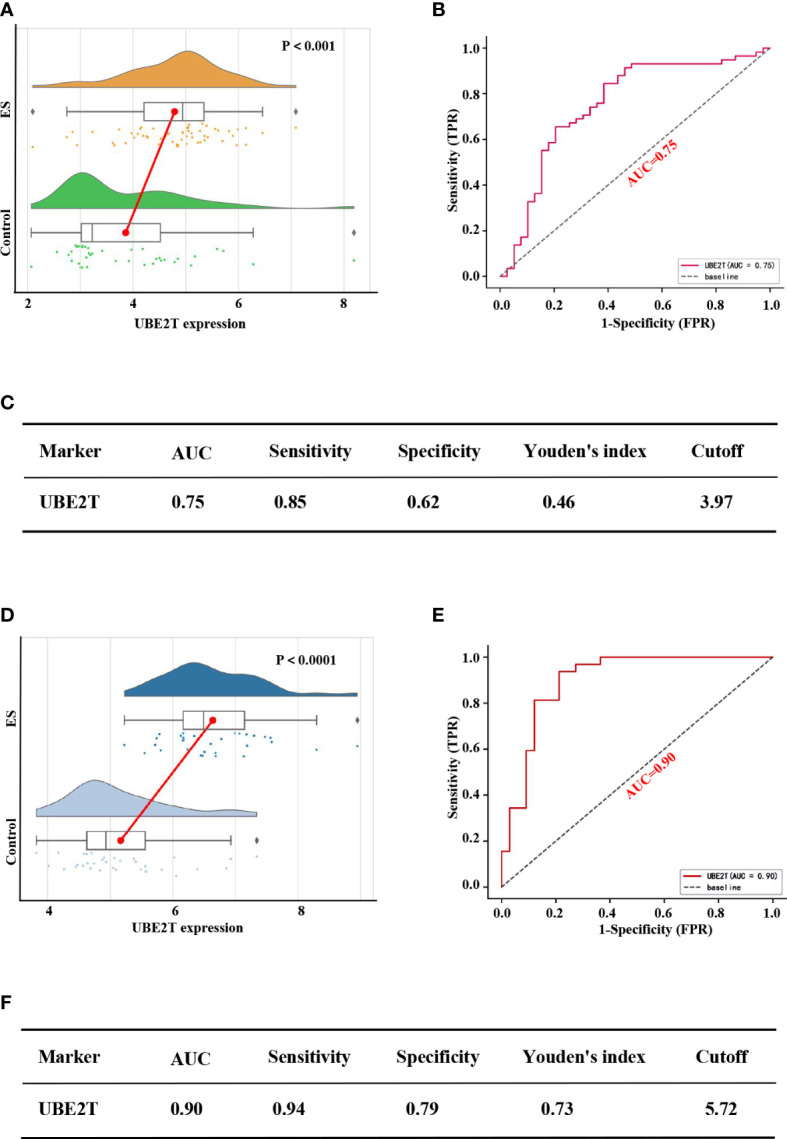
Performance of the core gene diagnostic ES in the training and verification sets. Based on the training set expression profiles (GSE17618 and GSE45544): **(A)** The difference in *UBE2T* expression between the ES and control groups. **(B)** The ROC curve of patients with ES based on the *UBE2T* gene. **(C)** The diagnostic value of the Core gene in distinguishing the ES group from the control group. According to the validation set expression profile (GSE68776): **(D)** The difference in *UBE2T* expression between the ES and control groups. **(E)** The ROC curve of people with ES based on the *UBE2T* gene. **(F)** The diagnostic value of the Core gene in distinguishing the ES group from the control group. AUC stands for Area Under the Curve; TPR stands for True Positive Rate; and FPR stands for False Positive Rate.

**Figure 5** depicted diagnostic value of *UBE2T* in ES. *UBE2T* was significantly overexpressed in ES in the training set (GES 17618 and GSE 45544) compared to the control group (*P*<0.001, **Figure 5A**). The Area Under Curve (AUC) of the ROC of *UBE2T* in diagnosing ES was 0.75, with sensitivity and specificity of 0.85 and 0.62, respectively (**Figures 5B, C**). Interestingly, the core gene is also excellent in the diagnostic evaluation of ES in the validation set (GSE68776). *UBE2T* expression was considerably increased in ES (*P*<0.0001, **Figure 5D**). The AUC of ROC was 0.90, its sensitivity was 0.94, and its specificity was 0.79 (**Figures 5E, F**). Obviously, these findings suggest that *UBE2T* had excellent value for ES diagnosis.

### 3.7 Analysis of MCODE-DEGs subgroups in ES

**Figure 6:**
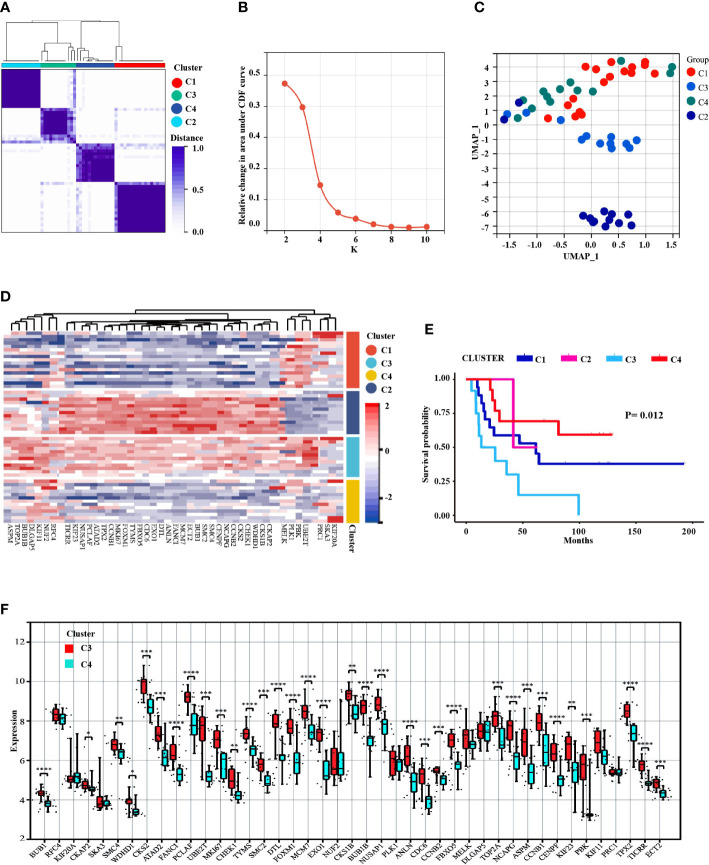
Prognosis identification of ES patients by clustering analysis based on MCODE-DEGs expression profifile. **(A)** Consensus clustering divided into 4 subgroups. **(B)** The CDF curve showed the consistency of clustering (K=2 the highest consistency, followed by K4). **(C)** UMAP dimension reduction analysis testified the classification. **(D)** The heat map displayed the different expression patterns of the MCODE-DEGs in 4 clusters. **(E)** The survival analysis of ES patients found significant differences among the four subgroups, *P*<0.05. **(F)** Boxplot revealed difference expression status of the MCODE-DEGs between C3 and C4 (*P<0.05, **P<0.01, ***P<0.001, ****P<0.0001).

55 samples of ES with patients (after removing non-conformance from inclusion criteria) were divided into 4 subgroups based on the expression levels of MCODE module genes: C1(N=17), C3(N=12), C4(N=13) and C2(N=13) (**Figure 6A**). Among the k = 2 to k = 10 clusters, K = 2 has the highest consistency, and k = 4 was the second (**Figure 6B** and **Supplement figure 1**). UMAP analysis indicated significant variations among the clusters (**Figure 6C**). Besides, The heat map revealed that the expression pattern of the MCODE-DEGs differed between the four subgroups (**Figure 6D**). In addition, Kaplan-Meier survival analysis showed significant differences in the subgroups (**Figure 6E**), especially C3 and C4, while ES patients with C3 experienced faster disease development than C4 patients. And, expression level of 34 genes in C3 (*UBE2T*, *PBK*, *CKAP2*, *CKS1B*, *WDHD1*, *CHEK1*, *CKS2*, *CCNB2*, *NCAPG*, *CENPF*, *SMC4*, *SMC2*, *BUB1*, *ECT2*, *MCM7*, *FANCI*, *ANLN*, *DTL*, *EXO1*, *CDC6*, *FBXO5*, *TYMS*, *FOXM1*, *MKI67*, *CCNB1*, *TPX2*, *ATAD2*, *PCLAF*, *NUSAP1*, *KIF23*, *TICRR*, *BUB1B*, *TOP2A* and *ASPM*) were considerably elevated compared to C4 (**Figure 6F**).

### 3.8 The correlation analysis between high expression of UBE2T and poor prognosis in ES patients

**Figure 7:**
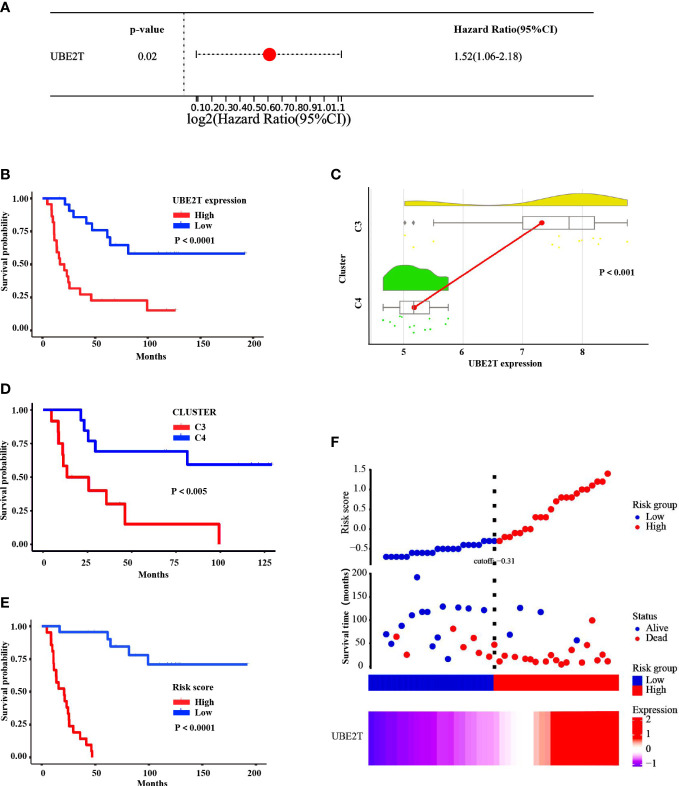
Correlation analysis between expression of *UBE2T* and prognosis in ES patients of the training cohorts. **(A)** HR and 95% CI of the core DEGs based on a unitvariable Cox regression analysis. **(B)** The Kaplan Meier survival curve demonstrated that the overall survival time of patients with *UBE2T* high expression was evidently shorter than low expression (*P*<0.0001). **(C)** The raincloud diagram showed the expression of *UBE2T* in C3 was significantly higher than C4 (*P*<0.001). **(D)** The K-M survival curve indicated that the survival time of C3 was significantly shorter than C4 (*P*<0.005). **(E)** The K-M survival curve displayed that patients in the high-risk score group had considerably lower survival time than the low-risk group (*P*<0.0001). **(F)** The distribution of risk score, survival status, and *UBE2T* expression level revealed that risk score increased as *UBE2T* expression increased, while survival rate decreased dramatically.

Based on training sets, a univariate Cox regression analysis was conducted to investigate the prognosis risk of core-DEGs in ES, and the results suggested that *UBE2T* was an independent risk factor (*P* **<**0.05, Hazard Ratio = 1.52, 95% CI) (**Figure 7A**). Besides, the Kaplan Meier survival curve demonstrated a connection between *UBE2T* expression and survival. The overall survival time of patients with high *UBE2T* expression was considerably shorter than low *UBE2T* expression (*P*<0.0001) (**Figure 7B**). In addition, the raincloud diagram showed that *UBE2T* in C3 was significantly higher than C4 (*P*<0.001) (**Figure 7C**), and the survival time was significantly shorter than that of C4 (*P*<0.005) (**Figure 7D**). These results suggest that the upregulation of *UBE2T* expression is associated with a worse outcome in ES patients. Furthermore, the study of risk score and survival time revealed that patients in the high-risk score group had considerably lower survival time than the low-risk group (*P*<0.0001) (**Figure 7E**). The findings indicated that the high-risk score group resulted in fast progression of disease. **Figure 7F** (including upper, middle, and lower parts) depicts the association between various risk scores, survival events, and gene expression changes. It can be shown that when *UBE2T* expression is up-regulated (**Figure 7F** lower part) and the risk score is increased (**Figure 7F** upper part), patients’ survival rates decline dramatically (**Figure 7F** middle part). As predicted, *UBE2T* was regarded as a risk independent factor, and risk scores increased as its expression rise.

## 4 Discussion

Ewing sarcoma, the second commonest malignant bony neoplasm and soft-tissue malignance neoplasm in kids and teenagers, is a serious threat to human life and health (1, 2), ES is and a highly aggressive tumor with nonspecific clinical features (2). Patients with standard risk and localized disease have a 70~80% survival, and patients with metastatic disease have an approximate 30% survival (23). Previously findings have suggested that the ES family of tumors is related to immunophenotypic characteristics, chromosomal translocation (such as extraosseous ES, peripheral primitive neuroectodermal neoplasm, Askin neoplasm (24), and FET-ETS gene fusion (25, 26). Although there has been improvement in the diagnosis of ES based on these preliminary studies, the specific pathogenesis remains largely unknown. Thus, it is urgent to ascertain novel biomarkers for this disease to enhance the efficiency of diagnosis and treatment. Microarray technology is beneficial for investigating genetic abnormalities for ES, which may be of benefit for the corroboration of novelty biomarkers to contribute to the improvement of early diagnosing and prediction prognosis for ES.

In the current investigation, two microarray datasets were selected from GEO, and bioinformatics analyzation was run to discover DEGs between ES tissues and nontumorous tissues. In all 187 DEGs were identified through analysis and comparison of those two datasets, including 56 downregulation genes and 131 upregulation. GO and KEGG enrichment analyzation were used to investigate interrelations in the DEGs. The up-regulation genes were majorly concentrated in cell dividing, mitosis nucleus dividing, proliferation, apoptotic process, response to drug, and positive regulation of apoptotic process, whereas this downregulation genes were primarily enriched in cell adhesion (**Table 3**). Life involves constant changes, and the cell cycle is required to maintain cell growth and DNA duplication, followed by cell division (mitosis), proliferation, and apoptosis. Remarkably, the cell cycle has an important effect on maintaining the normal process of life. Thus, dysregulation of the cell cycle process is closely related to the carcinogenesis or progression of tumors (27–29). In addition, recent reports have shown that the molecular mechanism of cell adhesion has a significant effect on collective cancer cell migrating, and mutations and changes in cell adhesion protein expression are frequently related to tumorous progression (30, 31). Whats more, changes in the tumor microenvironment may affect immune cell regulation (32). Our research findings revealed that, according to the Cytoscape ClueGO analysis, the biological processes of hub genes gathered in Mitosis cytokinesis, the dTMP biological process, and the positive regulation of self-antigen tolerance induction, which maintained stability of the cell cycle and the internal environment.

**Table 3:** Enrichment investigation of positive-regulation and negative-gulation genes with DEGs in ES specimens employing GO and KEGG Pathway.

Term	Description	Count	P value
Upregulation			
GO:0051301	cell division	21	8.53E-13
GO:0007067	mitotic nuclear division	16	3.43E-10
GO:0008283	cell proliferation	14	2.61E-06
GO:0006915	apoptotic process	13	8.67E-04
GO:0042493	response to drug	11	7.52E-05
GO:0043065	positive regulation of apoptotic process	10	3.44E-04
Hsa04110	cell cycle	9	1.50E-05
Hsa05166	HTLV-I infection	8	0.008161847
Downregulation			
GO:0007155	cell adhesion	9	7.67E-05
Hsa04514	cell adhesion molecules (CAMs)	4	0.015820009

Beside, In total, 15 hub genes were extracted relied on the most significant module with the degree rank (**Figure 4A**). One of these hub genes, ubiquitin-conjugating enzyme E2 T (*UBE2T*), catalyzes monoubiquitination, which is a significant posttranslational modification that affects a variety of biological activities, for instance, immune reactions, inflammation, cell proliferation, and cell differentiation (33–36). Interestingly, *UBE2T* plays an essential part in the DNA damage pathway, and it has been demonstrated to be correlated intimately with the development and poor prognosis of several cancers, such as gastric cancer, hepatocellular cancer, prostate cancer, and gallbladder cancer (37–40). Upregulation of *UBE2T* levels has been disclosed to enhance gastric cancer development through *RACK1* ubiquitination, and a novel powerful *UBE2T* inhibitor has been identified to suppress gastric cancer progression by blocking RACK1 ubiquitination after aberrant Wnt/β-catenin signaling (40). Moreover, Sun et al. (38) discovered that *UBE2T* was increased in HCC tissues, and that HCC sufferers with greater *UBE2T* quantities have a worse prognosis, demonstrating that *UBE2T*-regulated *H2AX* mono-ubiquitination may induce hepatocellular carcinoma radiation resistance by boosting *CHK1* activation. In addition, previous studies showed that the vulnerability of anticancer drugs is based on the involvement of proteins in ubiquitination and degradation, which provides a theoretical basis for the development of therapeutic drugs with genome modification (41, 42). As a result, *UBE2T* may be regarded as a therapeutic potential target for ES sufferers’ therapy.

However, there are few reports on the relationship between *UBE2T* and ES. Therefore, the present study analyzed several ES datasets in the GEO data bank and discovered that *UBE2T* expression was observed to be considerably greater in tumor samples than in nontumor samples. Furthermore, validation set and IHC findings displayed that the expression level of UBE2T was significantly higher in the sick tissues of Ewing’s sarcoma patients than the control group, and IHC analysis revealed that UBE2T was mostly expressed in the cytoplasm of Ewing’s sarcoma cells (**Figures 4E, F**). These results are consistent with our predictions. In addition, the investigation on the diagnostic value of core genes in ES observed that the AUC of *UBE2T* had excellent performance in both the training group and the verification group (**Figure 5**). Following that, we explored the relationship between the expression level of the *UBE2T* and prognosis by Cox regression and K-M survival analasis in ES patients according to the expression profiles of training sets. The findings revealed that *UBE2T* was an independent risk factor (**Figure 7A**), and patients with high expression of the *UBE2T* and the high-risk score, which led to a poor prognosis, had a negatively correlated survival time (**Figures 7B–E**). As a result, based on the above findings, this study demonstrated that *UBE2T* can be seen as an important value biomarker for diagnosis and treatment of ES, thereby providing a new potential therapeutic target for ES as well as an important new perspective for evaluating the effect of treatment and prognostic prediction.

## 5 Conclusion

In summary, the current examination found that *UBE2T* expression was greater in tumor tissues from ES patients than in non-tumor tissues and that *UBE2T* had an important value as a biomarker for the diagnosis of ES. Furthermore, increased *UBE2T* expression is associated with a terrible prognosis. As a result, *UBE2T* can be exploited as an independent prognostic biomarker in patients with ES. However, the existing research has drawbacks. First, consider the patient sample size constraints. As a consequence, *UBE2T* research should be added to the wider ES queue. Second, this study only investigated at *UBE2T* expression level in tumor tissues and did not researched *UBE2T* functionality *in vivo* or *in vitro*. As a result, further tests and investigations are required to uncover the potential mechanism of *UBE2T* in ES.

## Data availability statement

The datasets presented in this study can be found in online repositories. The names of the repository/repositories and accession number(s) can be found in the article/**Supplementary Material**.

## Author contributions

GQ: Collection, organizing, and review of the literature, preparing the manuscript, manuscript review and modification. YX and YQ: Samples collection, immunohistochemistry, bioinformatics analysis and revision of the manuscript. JQ and GC: Data processing and pictures arrangement. NZ and JD: Data analysis, editing of manuscript and revision. All authors read and approved the final manuscript. All authors contributed to the article and approved the submitted version.
