# Supplementary material for: Identification and validation of a novel ubiquitination-related gene UBE2T in Ewing’s sarcoma
Source: Front Oncol. 2023 Feb 16;13:1000949. doi: 10.3389/fonc.2023.1000949 (PMC9997212; doi:10.3389/fonc.2023.1000949)
Supplement: Supplementary file 1 [file DataSheet_1.pdf]

Supplementary Material

Supplementary Figure

A

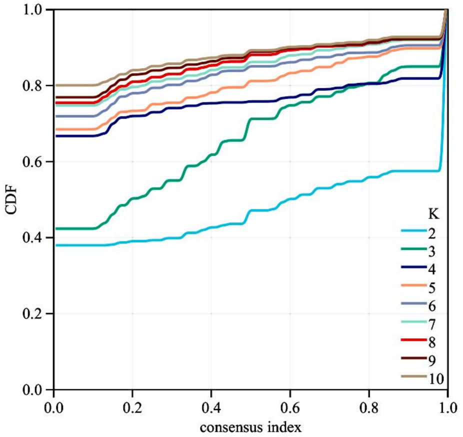

B

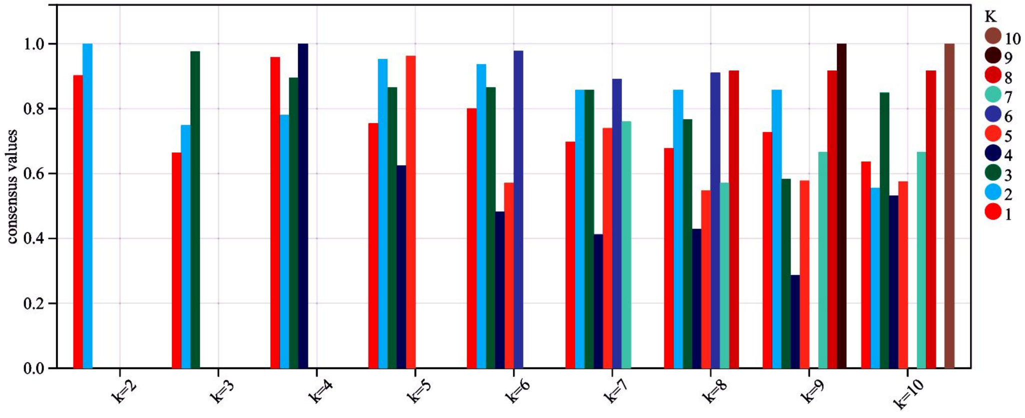

Supplementary Figure 1. Consensus clustering of MCODE-DEGs. (A) show the consensus clustering cumulativedistribution function of K=2 to K=10. (B) displays the number of clustersinside the group with the highest average consistency as K=2, while the number of clusters with the second highest consistency is K=4.
