# Supplementary material for: Identification and validation of a novel ubiquitination-related gene UBE2T in Ewing’s sarcoma
Source: Front Oncol. 2023 Feb 16;13:1000949. doi: 10.3389/fonc.2023.1000949 (PMC9997212; doi:10.3389/fonc.2023.1000949)
Supplement: Supplementary file 2 [file Table_1.docx]

1. Supplementary Data Figure 2 A

| **KEGG pathway** | **Count** | **PValue** | **Fold Enrichment** |
| --- | --- | --- | --- |
| hsa04110:Cell cycle | 9 | 2.00E-04 | 5.48661822 |
| hsa05150:Staphylococcus aureus infection | 5 | 0.005263316 | 6.999389499 |
| hsa04514:Cell adhesion molecules (CAMs) | 7 | 0.0104793 | 3.726435536 |
| hsa05145:Toxoplasmosis | 6 | 0.014306425 | 4.123276723 |
| hsa05166:HTLV-I infection | 9 | 0.017629696 | 2.678506533 |
| hsa04672:Intestinal immune network for IgA production | 4 | 0.023205791 | 6.433481412 |
| hsa04640:Hematopoietic cell lineage | 5 | 0.026781638 | 4.344448655 |
| hsa05200:Pathways in cancer | 11 | 0.031810733 | 2.115845986 |
| hsa04115:p53 signaling pathway | 4 | 0.056986491 | 4.5130392 |
| hsa04114:Oocyte meiosis | 5 | 0.05707035 | 3.405108405 |
| hsa05310:Asthma | 3 | 0.058122774 | 7.559340659 |
| hsa05140:Leishmaniasis | 4 | 0.065540828 | 4.25878347 |
| hsa05332:Graft-versus-host disease | 3 | 0.068810309 | 6.872127872 |
| hsa05169:Epstein-Barr virus infection | 5 | 0.075276664 | 3.098090434 |
| hsa04612:Antigen processing and presentation | 4 | 0.077011598 | 3.978600347 |
| hsa05330:Allograft rejection | 3 | 0.083964948 | 6.129195129 |
| hsa04068:FoxO signaling pathway | 5 | 0.098087861 | 2.8206495 |
| hsa05222:Small cell lung cancer | 4 | 0.099690496 | 3.557336781 |

1. Supplementary Data Figure 2 B

| **BP Term** | **Count** | **PValue** | **Fold Enrichment** |
| --- | --- | --- | --- |
| GO:0051301~cell division | 2.20E+01 | 1.06E-10 | 5.997142857 |
| GO:0007067~mitotic nuclear division | 1.70E+01 | 7.17E-09 | 6.540139296 |
| GO:0000281~mitotic cytokinesis | 7.00E+00 | 4.58E-07 | 23.02978056 |
| GO:0007062~sister chromatid cohesion | 9.00E+00 | 1.21E-05 | 8.336716681 |
| GO:0007155~cell adhesion | 1.70E+01 | 2.54E-05 | 3.533670034 |
| GO:0008283~cell proliferation | 1.50E+01 | 3.04E-05 | 3.910208644 |
| GO:0007094~mitotic spindle assembly checkpoint | 5.00E+00 | 4.85E-05 | 23.85227273 |
| GO:0000910~cytokinesis | 6.00E+00 | 1.38E-04 | 11.92613636 |
| GO:0006260~DNA replication | 9.00E+00 | 2.22E-04 | 5.539882698 |
| GO:0043065~positive regulation of apoptotic process | 1.20E+01 | 3.16E-04 | 3.816363636 |
| GO:0051726~regulation of cell cycle | 8.00E+00 | 3.20E-04 | 6.15542522 |
| GO:0007076~mitotic chromosome condensation | 4.00E+00 | 4.62E-04 | 25.44242424 |
| GO:0042127~regulation of cell proliferation | 9.00E+00 | 7.31E-04 | 4.641523342 |
| GO:0006915~apoptotic process | 1.60E+01 | 8.91E-04 | 2.692320026 |
| GO:0007077~mitotic nuclear envelope disassembly | 5 | 0.00111777 | 10.84194215 |
| GO:0042493~response to drug | 11 | 0.001346992 | 3.452302632 |
| GO:0031100~organ regeneration | 5 | 0.001433486 | 10.14990329 |
| GO:0001578~microtubule bundle formation | 4 | 0.002161745 | 15.26545455 |
| GO:0000086~G2/M transition of mitotic cell cycle | 7 | 0.003138588 | 4.874917054 |
| GO:0000082~G1/S transition of mitotic cell cycle | 6 | 0.004286116 | 5.612299465 |
| GO:0007059~chromosome segregation | 5 | 0.005527484 | 7.015374332 |
| GO:0001775~cell activation | 3 | 0.00558365 | 26.02066116 |
| GO:0044267~cellular protein metabolic process | 6 | 0.007886206 | 4.851309707 |
| GO:0007346~regulation of mitotic cell cycle | 4 | 0.008285005 | 9.540909091 |
| GO:0001666~response to hypoxia | 7 | 0.009339952 | 3.882928118 |

1. Supplementary Data Figure 2 C

| **MF Term** | **Count** | **PValue** | **Fold Enrichment** |
| --- | --- | --- | --- |
| GO:0005524~ATP binding | 34 | 2.19E-05 | 2.193804109 |
| GO:0005515~protein binding | 117 | 6.03E-05 | 1.284707375 |
| GO:0003682~chromatin binding | 13 | 7.58E-04 | 3.207204969 |
| GO:0043426~MRF binding | 3 | 0.001035025 | 57.87771429 |
| GO:0008017~microtubule binding | 9 | 0.001437409 | 4.173873626 |
| GO:0019901~protein kinase binding | 11 | 0.005704516 | 2.822051672 |

1. Supplementary Data Figure 2 D

| **CC Term** | **Count** | **PValue** | **Fold Enrichment** |
| --- | --- | --- | --- |
| GO:0000922~spindle pole | 11 | 1.47E-07 | 9.995213402 |
| GO:0016020~membrane | 48 | 3.10E-07 | 2.160948617 |
| GO:0005737~cytoplasm | 84 | 1.05E-06 | 1.59319268 |
| GO:0005829~cytosol | 60 | 3.08E-06 | 1.79264214 |
| GO:0005819~spindle | 10 | 3.67E-06 | 8.185411427 |
| GO:0005654~nucleoplasm | 52 | 8.61E-06 | 1.849950025 |
| GO:0000776~kinetochore | 8 | 1.71E-05 | 9.782071927 |
| GO:0005925~focal adhesion | 15 | 4.24E-05 | 3.799621928 |
| GO:0000940~condensed chromosome outer kinetochore | 4 | 5.38E-05 | 49.52173913 |
| GO:0030496~midbody | 8 | 3.28E-04 | 6.14223121 |
| GO:0070062~extracellular exosome | 47 | 4.16E-04 | 1.656009775 |
| GO:0000942~condensed nuclear chromosome outer kinetochore | 3 | 5.94E-04 | 74.2826087 |
| GO:0005634~nucleus | 76 | 7.17E-04 | 1.390083905 |
| GO:0005876~spindle microtubule | 5 | 9.76E-04 | 11.25494071 |
| GO:0000796~condensin complex | 3 | 9.83E-04 | 59.42608696 |
| GO:0015630~microtubule cytoskeleton | 7 | 0.002622091 | 5.060615678 |
| GO:0030027~lamellipodium | 7 | 0.005603481 | 4.333152174 |
| GO:0000307~cyclin-dependent protein kinase holoenzyme complex | 3 | 0.00966311 | 19.80869565 |
