# Supplementary material for: Identification and validation of a novel ubiquitination-related gene UBE2T in Ewing’s sarcoma
Source: Front Oncol. 2023 Feb 16;13:1000949. doi: 10.3389/fonc.2023.1000949 (PMC9997212; doi:10.3389/fonc.2023.1000949)
Supplement: Supplementary file 3 [file Table_2.doc]

**Supplementary Material --Figure4.**

GO and KEGG pathway enrichment analyses of the MCODE module of DEGs in ES samples.

| **Category** | **ID** | **Function Description** | **Count** | **P value** | **FDR** |
| --- | --- | --- | --- | --- | --- |
| BP | GO:0051301 | cell division | 16 | 1.81E-15 | 5.17E-13 |
| BP | GO:0007067 | mitotic nuclear division | 14 | 1.42E-14 | 2.03E-12 |
| BP | GO:0008283 | cell proliferation | 12 | 7.99E-10 | 7.62E-08 |
| BP | GO:0006260 | DNA replication | 8 | 8.61E-08 | 4.93E-06 |
| BP | GO:0051726 | regulation of cell cycle | 7 | 5.23E-07 | 2.49E-05 |
| BP | GO:0000086 | G2/M transition of mitotic cell cycle | 7 | 9.40E-07 | 3.84E-05 |
| BP | GO:0000281 | mitotic cytokinesis | 6 | 7.67E-09 | 5.48E-07 |
| BP | GO:0006974 | cellular response to DNA damage stimulus | 6 | 1.45E-04 | 0.002960183 |
| BP | GO:0006281 | DNA repair | 6 | 2.56E-04 | 0.004873601 |
| BP | GO:0000910 | cytokinesis | 5 | 5.51E-06 | 1.91E-04 |
| BP | GO:0007059 | chromosome segregation | 5 | 2.22E-05 | 5.78E-04 |
| BP | GO:0007062 | sister chromatid cohesion | 5 | 1.14E-04 | 0.002706386 |
| BP | GO:0007076 | mitotic chromosome condensation | 4 | 6.02E-06 | 1.91E-04 |
| BP | GO:0007094 | mitotic spindle assembly checkpoint | 4 | 1.50E-05 | 4.28E-04 |
| BP | GO:0007346 | regulation of mitotic cell cycle | 4 | 1.25E-04 | 0.002757975 |
| CC | GO:0005634 | nucleus | 31 | 2.84E-08 | 4.33E-07 |
| CC | GO:0005654 | nucleoplasm | 29 | 7.33E-14 | 4.31E-06 |
| CC | GO:0005737 | cytoplasm | 29 | 3.54E-07 | 4.47E-12 |
| CC | GO:0005829 | cytosol | 20 | 4.96E-05 | 2.62E-04 |
| CC | GO:0005819 | spindle | 9 | 2.92E-10 | 8.91E-09 |
| CC | GO:0005813 | centrosome | 9 | 4.91E-06 | 3.74E-05 |
| CC | GO:0000922 | spindle pole | 8 | 5.12E-09 | 1.04E-07 |
| CC | GO:0030496 | midbody | 7 | 4.76E-07 | 4.84E-06 |
| CC | GO:0005876 | spindle microtubule | 5 | 3.09E-06 | 2.70E-05 |
| CC | GO:0000776 | kinetochore | 5 | 3.56E-05 | 2.17E-04 |
| MF | GO:0005515 | protein binding | 36 | 7.65E-06 | 1.84E-04 |
| MF | GO:0005524 | ATP binding | 19 | 2.83E-09 | 2.04E-07 |
| MF | GO:0019901 | protein kinase binding | 10 | 2.29E-07 | 8.24E-06 |
| MF | GO:0003682 | chromatin binding | 8 | 3.86E-05 | 6.95E-04 |
| MF | GO:0008017 | microtubule binding | 6 | 1.41E-04 | 0.00203608 |
| KEGG | hsa04110 | Cell cycle | 8 | 5.22E-09 | 7.83E-08 |
| KEGG | hsa04114 | Oocyte meiosis | 5 | 1.01E-04 | 7.55E-04 |
| KEGG | hsa04914 | Progesterone-mediated oocyte maturation | 4 | 9.71E-04 | 0.004857351 |

**GO, Gene Ontology; BP, biological process; CC, cellular component; MF, molecular function; KEGG, Kyoto Encyclopedia of Genes and Genomes; DEGs, differentially expressed genes; FDR, false discovery rate**.
